# Investigation on the Injury Severity of Drivers in Rear-End Collisions Between Cars Using a Random Parameters Bivariate Ordered Probit Model

**DOI:** 10.3390/ijerph16142632

**Published:** 2019-07-23

**Authors:** Feng Chen, Mingtao Song, Xiaoxiang Ma

**Affiliations:** The Key Laboratory of Road and Traffic Engineering, Ministry of Education Tongji University, Shanghai 201804, China

**Keywords:** injury severity, rear-end crash, random parameter bivariate ordered probit

## Abstract

The existing studies on drivers’ injury severity include numerous statistical models that assess potential factors affecting the level of injury. These models should address specific concerns tailored to different crash characteristics. For rear-end crashes, potential correlation in injury severity may present between the two drivers involved in the same crash. Moreover, there may exist unobserved heterogeneity considering parameter effects, which may vary across both crashes and individuals. To address these concerns, a random parameters bivariate ordered probit model has been developed to examine factors affecting injury sustained by two drivers involved in the same rear-end crash between passenger cars. Taking both the within-crash correlation and unobserved heterogeneity into consideration, the proposed model outperforms the two separate ordered probit models with fixed parameters. The value of the correlation parameter demonstrates that there indeed exists significant correlation between two drivers’ injuries. Driver age, gender, vehicle, airbag or seat belt use, traffic flow, etc., are found to affect injury severity for both the two drivers. Some differences can also be found between the two drivers, such as the effect of light condition, crash season, crash position, etc. The approach utilized provides a possible use for dealing with similar injury severity analysis in future work.

## 1. Introduction

With the increase of vehicle miles/kilometers travelled, traffic crash has become one of the main factors that cause human injury and death along with huge property damage. According to the World Health Organization, nearly 1.25 million people died in traffic crashes worldwide annually and each fatality, on average, causes a loss of about 1.42 million dollars [1]. Injury crashes become a major concern of researchers, policy-makers, and the public [2]. There are various types of traffic crash such as rear-end, head-on, forward impact, side wipe, etc., among which rear-end crash results in a much great proportion of severe injuries or fatalities [3]. For instance, rear-end crashes account for 30% of all injuries and 29.7% of all property damage in the USA [4]. As one of the main concerns of the rear-end collision, drivers’ injury severity is influenced by many contributing factors, such as roadway alignments, environment characteristics, driver characteristics, traffic flow, etc. Accordingly, it is necessary to identify the factors related to rear-end crashes and having a comprehensive knowledge of these factors’ potential positive or negative effects is essential to prevent crashes and reduce injury severities [5].

To investigate the contributing factors of rear-end crash, vehicle laboratory crash test [6], numeric simulation [7,8], and field crash data analysis are three effective tools. As a direct and well-targeted method, vehicle laboratory crash test usually uses the scrapped vehicles and human body models to conduct vehicle collision tests. Such kinds of tests are non-repeatable and expensive; thus, fewer samples can be obtained. Conversely, the numerical simulation can be used to explore the injury mechanism by setting different parameter values and combinations of significant factors such as collision angle, impact speed, vehicle types, etc. The numerical simulation is a powerful tool; however, it needs many refined modeling and complex mechanics analysis, and some natural conditions are usually neglected.

As the crash data recorded in the real world become more and more detailed and specific, the field crash data analysis has been the most widely used method to analyze human injury severity in rear-end crashes. As pointed out by Chen et al. [9], these naturally collected data usually include almost all crash-related perspectives like human factors (age, seat position, sex, fatigue, alcohol usage, and so on), vehicle (speed, vehicle type, weight, and so on), and environment (light condition, road surface, weather, and so on). These data reflect the real state of crashes pretty well. Moreover, Wang et al. developed a quasi-vehicle-trajectory-based method to find crash contributing factors, presenting a possible use of real-time data [10]. Based on various methodological and analytical techniques in big data analysis, many prediction models are also obtained and applied, including multinomial logit models, latent class logit models, random parameter (mixed) logit models, ordered logit/probit models, support vector machine, and so on [11,12,13].

In terms of specific models for investigating injury severity in previous studies, there are also abundant researches. Yuan et al. developed a binary logistic regression model to predict occupant injury severity. This model identified corresponding affecting factors in rear-end crashes involving trucks as the front vehicle [14]. A multinomial logit model was utilized by Konstantina et al. to investigate severity outcomes of farm vehicle crashes and it was found that antiquated farm vehicles were more likely to suffer serious injuries or fatalities in crashes under insufficient light conditions [15]. Via the same approach, the corresponding factors were identified to classify four severity outcomes based on the most severe injuries in crashes [16]. Substantial difference between the impacts of variables on the driver-injury severity in single- and multi-vehicle accidents had been found by a mixed logit model developed by Chen and Chen [17].

Recently, some innovative methods have also been used in analyzing injury severity. Kunt et al. compared a genetic algorithm and an artificial neural network. The results demonstrated that the artificial neural network provides the best prediction [18]. In [3], Chen et al. studied the driver injury severity in rear-end crashes with the use of a multinomial logit model-Bayesian network hybrid approach, which performs reasonably well. The results indicated that the factors including inferior lighting conditions, windy weather, etc. could significantly increase driver injury severities in rear-end crashes. Other related researches can also be found and they present detailed insights into injury severity analysis [19,20,21,22,23,24,25,26,27].

Abundant as the models in assessing the injury severity seem, each model often has its own limitations. For instance, the genetic algorithm, artificial neural network, and Bayesian network have some deficiencies in interpreting the relationship between a certain factor and the injury severity. Moreover, injury severity has its natural character as severity outcomes are ordered from a low level to a high level naturally, and this should be noted in the model. After decades of exploration, the ordered probit model has been one of the most common approaches used in accident severity studies. In [28], Chandler et al. demonstrated an ordered probit model to examine the impact of various factors on injuries severity to passenger car occupants involved in truck-car collisions. Abdel-Aty developed several ordered probit models to examine driver injury severity for crashes at roadway sections, signalized intersections, and toll plazas in Central Florida, respectively [29]. Alcohol, lighting conditions, and the existence of a horizontal curve were found to be of significance in the roadway sections’ model. An ordered probit model was developed to analyze the injury severity of wrong-way driving crashes, which also found the lighting condition to be significant to injury severity [30].

In this paper, instead of the most severe injury, we focused mainly on comparing the injury severity of the two drivers in passenger cars involved in the same rear-end crash. There exists a problem if we simply use the ordered probit model, since the two drivers share some common conditions such as road surface condition, but the model allows only one dependent variable. Moreover, there may be some unobserved factors causing correlation. To address this correlation, the bivariate ordered probit model was proposed and two dependent variables were designed for each crash. Furthermore, compared with the fixed-parameter model, the random-parameter regression model is often adopted, because the unobserved heterogeneity issues can be addressed by allowing parameters to vary across observations [2,5,31].

The remainder of this paper is organized as follows: in Section 2, the random parameter bivariate ordered probit model adopted in this paper is introduced in detail; in Section 3, a brief description of the rear-end crashes is provided; Section 4 presents the results and discussion of the model; Finally, Section 5 provides the summaries and conclusions of the work.

## 2. Methodology

As previously stated, the injury severity levels of two drivers involved in the same rear-end crash are typically correlated considering they usually share the same lighting condition, road alignment, road surface condition, and, especialy, other unobserved factors. To address such kinds of possible correlation problems, the bivariate ordered probit model, which is a hierarchical system of two equations, can be employed to model a simultaneous relationship of two response variables [22].

Suppose $y_{ij}$ is the observed injury severity; (*i, j*) is the index indicating the two drivers involved in the same rear-end crash, where *i* (*i* = 1, 2, 3, …, n) refers to the crash number and j refers to the driver number in a certain crash *i* (*j* = 1 for the rear vehicle, 2 for the front vehicle). Thereafter, the latent (unobserved) injury severity propensities of the two drivers match their actual injury severity, as presented in the following equations [7]:

(1) $$y_{i,j\;=\;1}=k,\;\;\;if\;\mu_{j\;=\;1,\;k-1}<y_{i,\;\;j=1}^{*}<\mu_{j\;=\;1,\;k}$$

(2) $$y_{i,j\;=\;2}=l,\;\;\;\;if\;\mu_{j\;=\;2,l-1}<y_{i,\;\;j=2}^{*}<\mu_{j\;=\;2,\;l}$$

where $\mu_{j,k-1},\mu_{j,k},\mu_{j,l-1},\mu_{j,l}$ are thresholds or cut-off values used to determine observed injury severity levels of both two drivers, their values are relative to their corresponding injury factors in crash i. Additionally, *k* (*k* = 0, 1, 2, …, *K*) and *l* (*l* = 0, 1, 2, …, *L*) represent ordinal categories of injury severity sustained by each driver. $y_{i,\;\;j\;=\;1}^{\;*}$ and $y_{i,\;\;j=2}^{\;*}$ can be calculated using real data as follows:

(3) $$y_{i,\;\;j=1}^{*}=\beta_{0,j=1}^{\prime }+\beta_{1,j=1}^{\prime }*x1_{i,j=1}+\beta_{2,j=1}^{\prime }*x2_{i,j=1}+\ldots +\varepsilon_{i,j=1}=\beta_{1}^{\prime }X_{i,j=1}+\varepsilon_{i,j=1}$$

(4) $$y_{i,\;\;j=2}^{*}=\beta_{0,j=2}^{\prime }+\beta_{1,j=2}^{\prime }*x1_{i,j=2}+\beta_{2,j=2}^{\prime }*x2_{i,j=2}+\ldots +\varepsilon_{i,j=2}=\beta_{2}^{\prime }X_{i,j=2}+\varepsilon_{i,j=2}$$

where $X_{ij}$ is the variable vector; $\beta_{j}^{\prime }$ is a parameter vector remaining to be estimated; $\varepsilon_{ij}$ represents the random components that capture all unobserved factors associated with two involved parties, which is assumed to follow a bivariate normal distribution as follows:

(5) $$\left(\begin{array}{c} \varepsilon_{i,j=1}\\ \varepsilon_{i,j=2} \end{array}\right)\sim N\left[\left(\begin{array}{c} 0\\ 0 \end{array}\right),\;\left(\begin{array}{c} 1\;\;\rho \\ \rho \;\;1 \end{array}\right)\;\right]$$

where ρ is the estimated correlation parameter between $\varepsilon_{i,j\;=1}$ and $\varepsilon_{i,j=2}$. Therefore, the joint probability when the injury severities of two drivers in a rear-end crash are k and l, which, respectively, can be expressed as follows:

(6) $$\begin{array}{l} \mathrm{Pr}\left(y_{i,j=1}=k;\;y_{i,j=2}=l\right)\\ \;\;\;\;\;\;\;\;=\mathrm{Pr}\left(\mu_{j=1,\;k-1}<y_{i,\;\;j=1}^{*}<\mu_{j=1,\;k};\;\mu_{j=2,l-1}<y_{i,\;\;j=2}^{*}<\mu_{j=2,\;l}\right)\\ \;\;\;\;\;\;\;\;=\mathrm{Pr}\left(\mu_{j=1,\;k-1}<\beta_{1}^{\prime }X_{i1}+\varepsilon_{i1}<\mu_{j=1,\;k};\;\mu_{j=2,l-1}<\beta_{2}^{\prime }X_{i2}+\varepsilon_{i2}<\mu_{j=2,\;l}\right)\\ \;\;\;\;\;\;\;\;=\mathrm{Pr}\left(\mu_{j=1,\;k-1}-\beta_{1}^{\prime }X_{i1}<\varepsilon_{i1}<\mu_{j=1,\;k}-\beta_{1}^{\prime }X_{i1};\;\mu_{j=2,l-1}-\beta_{2}^{\prime }X_{i2}<\varepsilon_{i2}<\mu_{j=2,\;l}-\beta_{2}^{\prime }X_{i2}\right)\\ \;=\Phi_{2}\left(\mu_{j=1,\;k}-\beta_{1}^{\prime }X_{i1},\mu_{j=2,\;l}-\beta_{2}^{\prime }X_{i2};\rho \right)-\Phi_{2}\left(\mu_{j=1,\;k-1}-\beta_{1}^{\prime }X_{i1},\mu_{j=2,\;l}-\beta_{2}^{\prime }X_{i2};\rho \right)\\ \;-\Phi_{2}\left(\mu_{j=1,\;k}-\;\;\;\;\;\;\;\;\;\;\;\beta_{1}^{\prime }X_{i1},\mu_{j=2,\;l-1}-\beta_{2}^{\prime }X_{i2};\rho \right)\\ \;+\Phi_{2}\left(\mu_{j=1,\;k-1}-\beta_{1}^{\prime }X_{i1},\mu_{j=2,\;l-1}-\beta_{2}^{\prime }X_{i2};\rho \right) \end{array}$$

where *Φ*_2_( ) is the standard bivariate normal cumulative distribution function.

Due to the characteristic of the bivariate ordered probit model, the signs of the parameters are of great interest. A positive sign of the parameter indicates a positive effect on the probability of the injury severity.

While bivariate ordered probit can address the problem of factors correlation between two drivers involved in the rear-end crash, this method assumes the parameters $\beta_{1}^{\prime },\;\beta_{2}^{\prime }$ to have a certain value neglecting the effect of unobserved heterogeneity of observations. This constraint on model parameters may lead to inconsistent and biased parameter estimation [2]. As previously stated, the random-parameter method can address the unobserved heterogeneity by allowing the parameters to vary across observations. Therefore, the random parameters bivariate ordered probit model can be derived by setting:

(7) $$\beta_{i}^{\prime }=\beta +\gamma_{i}$$

where $\beta_{i}^{\prime }$ is the vector of specific parameters; $\gamma_{i}$ is the randomly distributed term which is normally distributed with a zero mean value and variance $\sigma^{2}$.

## 3. Data Description

In this paper, the crash data from the National Automotive Sampling System (NASS) General Estimates System (GES) were obtained. Data for all car-car rear-end collisions occurring from the calendar year 2011 to 2015 were sampled as the basic dataset. These crash data recorded details of the crash time, driver information, environmental condition, roadway condition, vehicle characters, etc. Some data were excluded for those crashes in which the driver’s injury severity was unknown or serious errors and missing existed.

The final dataset included a total of 15,159 rear-end crashes involving 30,318 motor vehicle drivers. The original injury severity was coded on the KABCO scale and was regrouped due to the low proportion of fatal injuries and incapacitating injury levels. The new groups were presented as follows:

- L1: No injury (O) and no apparent injury (O);
- L2: Possible injury (C);
- L3: Non-incapacitating evident injury (B), suspected minor injury (B);
- L4: Incapacitating injury (A), suspected serious injury (A), fatal injury (K).

As previously stated, the differences and similarities of the two drivers in the same rear-end crash should be addressed. When analyzing the crash data, drivers were separated into two groups: the front vehicle and the rear vehicle. Table 1 presents a summary of the joint distribution of the injury severity for drivers in front vehicles and drivers in rear vehicles. It can be found that the distributions of injury severity for drivers in front vehicles and rear vehicles vary in some degrees. For instance, 33.4% of the drivers in front vehicles tend to suffer an injury while the value is 19.4% for rear vehicles. Moreover, the proportions of L2, L3, L4 for rear vehicles are all smaller than those of front vehicles. This indicates that the drivers in front vehicles seem to suffer more serious injury.

**Figure 1:**
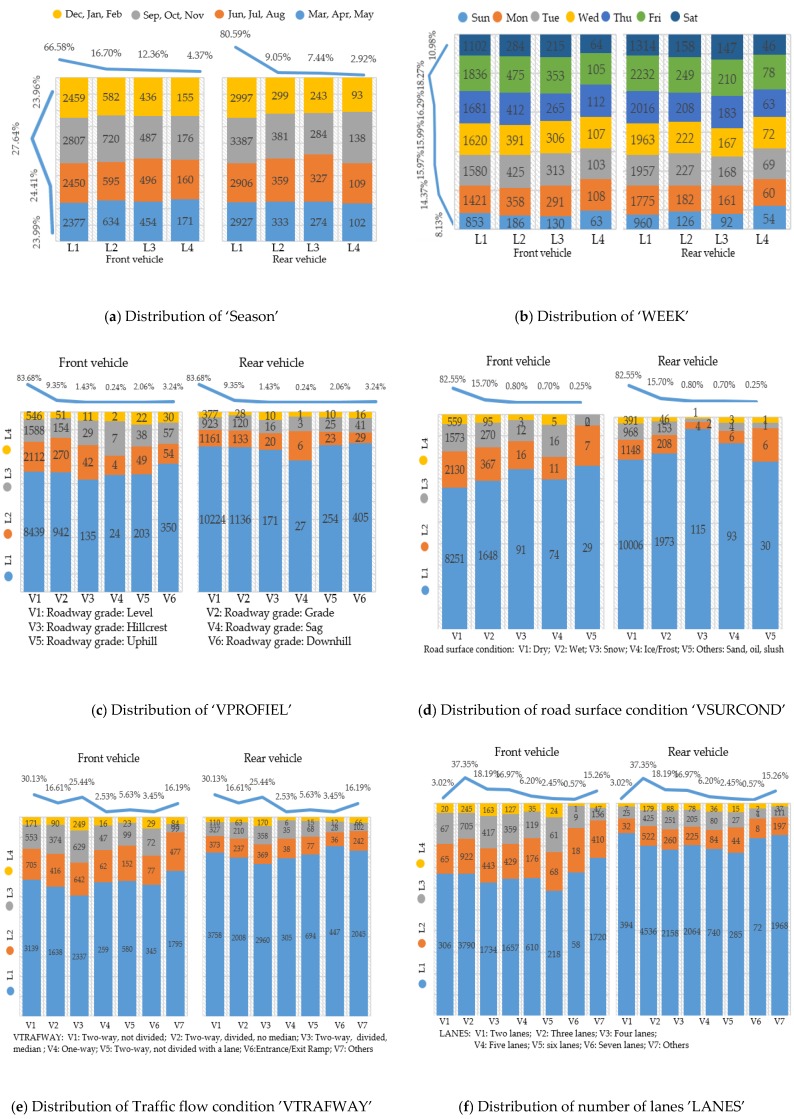
Summary statistics for driver-related, vehicle-related and other independent variables.

A large number of explanatory variables were examined and possible related variables are presented in Table 2 and Figure 1 (some variables with complex classifications are depicted in Figure 1, the percentage curves are also presented). It is interesting to note some substantial differences between the two drivers. For instance, the drivers in rear vehicles tend to have a higher proportion of alcohol involved and drug use. Since prior work has identified many injury-related variables, investigation on the differences and similarities between the two drivers involved in the same rear-end crash needs further efforts and the quantification of variables’ effect on injury severity needs to be addressed by the statistical model.

To improve the process of modeling, the explanatory variables were coded into dummy variables (0/1) when conducting model estimations. For instance, the crash season can be described using three binary indicates (1,0,0 for Spring; 0,1,0 for Summer; 0,0,1 for Autumn and 0,0,0 for Winter).

## 4. Model Estimation and Discussion of Results

To start the estimation process of the random parameter bivariate ordered probit model, the initial value of variable coefficients had to be set up. Therefore, two independent ordered probit models were developed respectively after separating the dataset into two parts (one for the driver in the front vehicle and another for the driver in the rear vehicle). All variables that were significant at a 90% confidence level in two separate models were remained for the subsequent analysis of random parameters bivariate ordered probit model.

Table 3 presents the results of the random parameters bivariate ordered probit models. Parameter estimates, *p*-values, standard errors, and z value of the estimates were included. Compared with the results of two separately ordered probit models, substantive improvements can be found in the random parameters bivariate ordered probit model, such as the significance level of variables and the z value. This indicates that the random parameters bivariate ordered probit model indeed addressed the issue of correlation between the outcomes in the same crash and the unobserved heterogeneity across the observations.

As presented in Table 3, the final model includes all significant variables and some of them are set as fixed parameters or random parameters according to constant estimation (the parameter without a significant standard deviation is set as a fixed parameter). The value of ‘Percent observations’ indicates the distribution of the value of random parameter (above zero and below zero, respectively), which is determined by the value of mean and standard deviation.

From Table 3, the coefficient value of younger drivers (age 24 and below) was negative for both the two parties indicating that younger drivers generally sustained lower level of injury severity. This result was consistent with previous studies of Abay et al. and Chiou et al. and this may be because of the physiological or driving behavior differences such as reaction time [32,33]. Moreover, drivers in the rear vehicle at an age between 25 and 63 were found to suffer a lower level of injury severity, while the result of drivers in the front vehicle went in the opposite way.

As for the driver’s gender, male drivers in both two vehicles seemed to suffer less severe injuries. However, the effect of gender seemed to be mixed as the male driver had been found to be less injured in a previous work by Russo et al. [11] and more at risk for injuries in another work by Chen et al. [34]. In fact, the ‘Percent observations’ of driver gender may be used to account for this mixed effect as coefficient’s value has a distribution of 20.05% versus 79.95% (above zero versus below zero) for the front vehicle, 14.01% versus 85.99% for the rear vehicle. It was also found that drivers tended to sustain more severe injuries when the airbag was not deployed or the seat belt was not in use. This is consistent with common sense and highlights the importance of seat belt and airbag checking (occupant protection systems).

With positive coefficient values, injury severities tended to increase with alcohol or drug use by the driver in the rear vehicle. However, this effect had not been found for the driver in front vehicle. This difference may be explained as follows: drivers who were involved in alcohol or drug were usually found to drive at a relatively higher speed, and speeding was one of the main causes of rear-end crashes with severe injuries.

From the aspect of vehicle information, the front vehicle whose manufacturer’s model year was before 2001 (1995 and before, from 1996 to 2000) and the rear vehicle whose manufacturer’s model year was before 1996 (1995 and before) were more likely to bring the drivers a serious injury. This effect had been found in a previous study, which showed that old vehicles had a significantly greater chance of being involved in an injury crash [35]. This may be explained by the lack of fine maintenance and advanced safety design on old vehicles. This factor has a mixed effect on injury severity for the rear vehicle according to the value of ‘Percent observations’.

Interesting results can be found regarding the effect of crash time. For drivers in the front vehicles, the injury severity of crashes that happened on Mondays tended to be higher. For drivers in the rear vehicles, more severe injuries might be found in crashes happened on a summer day. For both front and rear vehicles, drivers tended to suffer a severe injury during nighttime (beyond 7:00–18:00). Related conclusions about time factors’ effect on injury severity had also been obtained in previous studies. For instance, lower level of injury had been found in the crashes during the winter month and relative researches attributed this to lower speed and more cautious drivers on a winter day [11]; this may explain why lower injury severity would be sustained by drivers in rear vehicles on a snowy road surface condition as shown in Table 3. Summer season and nighttime had been found to be significantly associated with the injury severity, causing a positive effect on the possibility of the injuries [5]. Moreover, both drivers seemed to suffer a more severe injury when light level that existed at the time of the crash is daylight.

The traffic way related factor (VTRAFWAY), which focused on the value of coefficients only, it can be concluded that the level of injury severity for both the two drivers in rear-end crashes may increase under a two-way divided traffic flow. Moreover, the injury severity for drivers in front vehicles may also become more serious at the entrance or exit ramp where the sight distance is usually limited. As for the influence of traffic flow, drivers in rear vehicles may suffer a more severe injury at the one-way traffic flow. Interestingly, this factor’s effect is not absolutely positive or negative for the front vehicle according to the result of ‘Percent observations’ for this parameter. It is much better to say the effect is more possible to be positive (over a 50% probability).

For lane-related factors, the injury outcome of drivers in rear vehicles tends to be higher within a straight lane. The crash position reflected the regional characteristics of the crashes and the crash position had also been found to be related to the injury severity as presented in Table 3. A converse effect of crash position was shown on two drivers as the drivers in front vehicles may sustain a more severe injury in the Midwest of the country and the drivers in rear vehicles may suffer a lower level of injury in the Midwest and South of the country (the classification of region can be found in GES Analytical User’s Manual, for instance, Midwest includes OH, IN, IL, MI, WI, MN, ND, SD, NE, IA, MO, and KS of the United States). This phenomenon indicates that there may be a region difference on injury severity of the rear-end crash.

Apart from addressing the issue as to unobserved heterogeneity across observations, the random parameter bivariate ordered probit models also quantifies the correlation in the error terms between the models for each pair of crash-involved drivers by estimating the correlation parameter (𝜌). This index actually reflects the correlation in unobserved factors affecting injury severity in each rear-end crash, which may not be included in the recorded data. As presented in Table 3, the value of 𝜌 is positive suggesting that unobserved factors tend to jointly increase (or decrease) the level of injury sustained by two drivers involved in the same crash.

## 5. Conclusions

Rear-end crashes have become one of the main factors of human injury along with huge property damage; thus, it is necessary to figure out the possible cause of such kind of traffic accidents. This study examined the degree of injury sustained by drivers involved in the car-car rear-end crashes and the significant factors affecting injury severity. To address the within-crash correlation between the two drivers involved in the same crash and varying effects across observations, a random parameters bivariate ordered probit model was developed concerning the ordered probit model. Specifically, this model used two equations to describe injury severity for two drivers and proposed a correlation parameter to demonstrate correlation in unobserved factors between two drivers. The value of the correlation parameter suggests that these unobserved factors throw a joint increase (or joint decrease) effect on the degree of injury severity of the two drivers in the same crash. This indicates it is necessary to account for this within-crash correlation in model developing instead of heterogeneity in parameter effects only.

Specific effects of all significant variables on the injury for two drivers were also investigated and presented in this paper. For both vehicles, older age and female drivers tended to sustain a greater injury in rear-end crashes. The injury severity level with airbag or seat belt not in use becomes relative higher indicating the importance of a guaranteed occupant protection system. Furthermore, an older vehicle, nighttime, two-way traffic, and a light condition of daylight also caused positive effects on the injury severity for both two drivers. Although many common variables and consistent effects can be found, there are also some differences between the two drivers. For the drivers in rear vehicles, alcohol or drug use show positive effects on injury severity suggesting a necessary restrict on drivers’ alcohol or drug use. However, this effect has not been found on drivers in front vehicles. The outcome of injury for drivers in rear vehicles in a summer day or on a snowy road surface also increased, indicating a time-relation with the crash injury. Similar results have been presented about these effects on injury severity in previous studies [5,11,32,33,34]. One interesting result is that converse effects by crash position on two drivers can be found: when crashes occur in Midwest of the USA, drivers in front vehicles are prone to suffer a more severe injury, while drivers in rear vehicles sustain less injury. It has to be addressed that the paper presents the ‘Percent observations’ for random parameters, which may be used to explain the mixed effect for a certain factor.

Since rear-end crashes are related to many factors, this study gives an insight into several significant variables. Abundant as the rear-end crash data seem, the outcome of the current study is still limited with a certain dataset, which may cause a certain over-estimation. Exhaustive as the consideration of possible variables is, some potentially crucial information such as traffic volume has been neglected due to the lack of available data. However, the outcome of this study can still be used in some possible aspects like developing effective driver training, setting vehicle’s using year, offering risk information on road management, etc. There are also several promising extending of this work, e.g., examining different collision type, addressing temporal correlation in injury severity, or the injury of occupants in the same vehicle.

## Figures and Tables

**Table 1:** Summary of injury severity for rear-end crash-involved drivers.

Injury Severity of Driver in the Front Vehicle	Injury Severity of Driver in the Rear Vehicle				Total
	L1	L2	L3	L4	
No injury (L1)	8723	576	585	209	10,093 (66.6%)
Possible injury (L2)	1683	548	220	80	2531 (16.7%)
Non-incapacitating injury (L3)	1374	188	264	47	1873 (12.3%)
Incapacitating or fatal injury(L4)	437	60	59	106	662 (4.4%)
Total	12,217 (80.6%)	1372 (9.0%)	1128 (7.4%)	442 (2.9%)	15,159 (100%)

**Table 2:** Summary statistics for driver-related, vehicle-related and other independent variables.

Variables	Description and Classification	Driver in the Front Vehicle				Driver in the Rear Vehicle				Total
		L1	L2	L3	L4	L1	L2	L3	L4	
HOUR	Day: 7:00~18:00	8437	2098	1533	472	10,193	1130	890	327	25,080
	Night: else	1656	433	340	190	2024	242	238	115	5238
AGE	Age 24 and below	2303	433	310	87	4632	438	366	131	8700
	Age between 25 and 63	6770	1851	1395	499	6673	790	631	258	18,867
	Age 64 and above	1020	247	168	76	912	144	131	53	2751
SEX	Male	4827	952	637	267	6312	574	454	188	14,211
	Female	5266	1579	1236	395	5905	798	674	254	16,107
ALCOHOL	Alcohol involved	22	8	4	8	275	50	48	37	452
	Alcohol not involved	10,071	2523	1869	654	11,942	1322	1080	405	29,866
DRUGS	Drugs use	7	1	2	4	70	20	26	23	153
	No drugs use	10,086	2530	1871	658	12,147	1352	1102	419	30,165
AIR_BAG	Airbag not deployed	260	157	165	124	1896	588	527	174	3891
	Airbag deployed	9833	2374	1708	538	10,321	784	601	268	26,427
REST_USE	Seat belt equipment not in use	62	35	24	17	104	30	52	51	375
	Seat belt equipment in use	10,031	2496	1849	645	12,113	1342	1076	391	29,943
VALIGN	Roadway alignment: Straight	9539	2440	1787	620	11,547	1326	1092	421	28,772
	Curve	554	91	86	42	670	46	36	21	1546
REGION	Northeast	2164	642	90	142	2473	357	104	104	6076
	Midwest	1797	313	512	139	2306	142	225	88	5522
	South	4971	1155	1122	339	6074	682	625	206	15,174
	West	1161	421	149	42	1364	191	174	44	3546
SPEEDREL	Driver’s speed related	109	17	11	10	2492	334	281	115	3369
	Driver’s speed not related	9984	2514	1862	652	9725	1038	847	327	26,949
LGTCON	Daylight	7785	1944	1444	443	9373	1067	863	313	23,232
	Dark	1954	514	377	191	2431	256	231	118	6072
	Dawn or Dusk	354	73	52	28	413	49	34	11	1014
WEATHER	Clear	7164	1836	1271	482	8627	970	822	334	21,506
	Rain	1154	254	193	68	1396	146	95	32	3338
	Snow, Freezing Rain or Drizzle	135	33	24	9	174	11	10	6	402
	Fog, Smog, Smoke and so on	25	8	3	0	30	3	3	0	72
	Cloudy	1615	400	382	103	1990	242	198	70	5000
INT_HWY	Crash occurred on an interstate way	1442	360	299	169	1765	198	192	115	4540
	Crash not occurred on an interstate way	8651	2171	1574	493	10,452	1174	936	327	25,778
MDLYR	Manufacturer’s model year: before 1995	365	125	103	39	873	100	119	30	1754
	1996–2000	1113	361	322	87	2303	299	208	85	4778
	2001–2005	2400	669	494	205	3662	406	339	123	8298
	2006–2010	3726	818	594	193	3579	380	296	137	9723
	2011–2015	2489	558	360	138	1800	187	166	67	5765
BDYTYP	The car has four doors	7152	1857	1373	491	8834	1013	787	129	21,636
	The number of car’s doors is not four	2941	674	500	171	3383	359	341	313	8682
WRKZONE	Within the boundaries of a work zone	191	40	46	12	228	19	28	14	578
	Not within the boundaries of a work zone	9902	2491	1827	650	11,989	1353	1100	428	29,740

**Table 3:** Results of random parameter bivariate ordered probit models.

Variables	Coefficient	Standard Error	Z Value	Percent Observations	
				Above 0	Below 0
**Driver in the Front Vehicle**					
Constant	−0.2028 ***	0.0677	−3.0	—	—
▲ AGE: ≤24	−0.3182 ***	0.0269	−11.8	2.74%	97.26%
Standard deviation	0.1657 ***	0.0238	7.0	—	—
▲ SEX: Male	−0.4426 ***	0.0223	−19.9	20.05%	79.95%
Standard deviation	0.5292 ***	0.0177	29.8	—	—
▲ AIRBAG: Not deployed	0.8767 ***	0.0449	19.5	99.95%	0.05%
Standard deviation	0.2692 ***	0.0432	6.2	—	—
▲ SEAT BELT: Not in use	0.5594 ***	0.1025	5.5	—	—
SPEEDREL: Speed related	−0.3872 ***	0.1163	−3.3	—	—
▲ MDLYR: 2001–2005	−0.1392 ***	0.0320	−4.3	7.08%	92.92%
Standard deviation	0.0945 ***	0.0203	4.7	—	—
▲ MDLYR: 2006–2010	−0.3648 ***	0.0307	−11.9	0.96%	99.04%
Standard deviation	0.1559 ***	0.0178	8.8	—	—
▲ MDLYR: 2011–2015	−0.3569 ***	0.0331	−10.8	0.23%	99.77%
Standard deviation	0.1255 ***	0.0216	5.8	—	—
BDYTYP: Four doors	0.0839 ***	0.0232	3.6	76.42%	23.58%
Standard deviation	0.1164 ***	0.0121	9.6	—	—
WEEK: Monday	0.0592 **	0.0291	2.0	—	—
▲ HOUR: 7:00~18:00	−0.1753 ***	0.0370	−4.7	0.69%	99.31%
Standard deviation	0.0713 ***	0.0114	6.3	—	—
▲ VTRAFWAY: Two-way, divided no median	0.1414 ***	0.0292	4.9	68.44%	31.56%
Standard deviation	0.2967 ***	0.0242	12.3	—	—
VTRAFWAY: Entrance/Exit Ramp	0.0971 *	0.0588	1.7	57.53%	42.47%
Standard deviation	0.4995 ***	0.0598	8.4	—	—
REGION: Midwest	0.0876 ***	0.0273	3.2	56.75%	43.25%
Standard deviation	0.5032 ***	0.0257	19.6	—	—
▲ Light Condition: Daylight	0.1237 **	0.0577	2.2	100%	0
Standard deviation	0.0306***	0.0118	2.6	—	—
Light Condition: Dark	0.1215 **	0.0608	2.0	—	—
$\mu_{1}$	0.6191 ***	0.0113	54.8	—	—
$\mu_{2}$	1.4814 ***	0.0202	73.3	—	—
**Driver in the rear vehicle**					
Constant|	−0.7899 ***	0.0921	−8.58	0	100%
Standard deviation	0.2067 ***	0.0123	16.78	—	—
▲ AGE: ≤24	−0.4894 ***	0.0454	−10.79	8.85%	91.15%
Standard deviation	0.3615 ***	0.0221	16.36	—	—
AGE: 25–63	−0.2780 ***	0.0426	−6.53	0	100%
Standard deviation	0.0754 ***	0.0166	4.54	—	—
▲ SEX: Male	−0.3822 ***	0.0261	−14.67	14.01%	85.99%
Standard deviation	0.3554 ***	0.0192	18.49	—	—
ALCOHOL: Involved	0.2452 ***	0.0688	3.56	—	—
DRUGS: Involved	0.5898 ***	0.1110	5.31	81.06%	18.94%
Standard deviation	0.6645 ***	0.1128	5.89	—	—
▲ AIRBAG: Not deployed	1.0486 ***	0.0267	39.3	—	—
▲ SEAT BELT: Not in use	1.1682 ***	0.0818	14.28	91.77%	8.23%
Standard deviation	0.8390 ***	0.0906	9.27	—	—
MDLYR: 1995–2000	−0.1192 **	0.0531	−2.25	6.18%	93.82%
Standard deviation	0.0774 ***	0.0281	2.75	—	—
▲ MDLYR: 2001–2005	−0.1669 ***	0.0506	−3.3	17.36%	82.64%
Standard deviation	0.1782 ***	0.0226	7.88	—	—
▲ MDLYR: 2006–2010	−0.2833 ***	0.0517	−5.48	25.78%	74.22%
Standard deviation	0.4353 ***	0.0244	17.81	—	—
▲ MDLYR: 2011–2015	−0.2049 ***	0.0554	−3.70	—	—
SEASON: Jun, Jul, Aug	0.0933 ***	0.0291	3.20	94.74%	5.26%
Standard deviation	0.0575 **	0.0246	2.34	—	—
▲ HOUR: 7:00~18:00	−0.2304 ***	0.0451	−5.11	—	—
VSURCOND: Snow	−0.6991 ***	0.2430	−2.88	—	—
Standard deviation	0.5531 **	0.2344	2.36	—	—
▲ VTRAFWAY: Two-way, divided no median	0.1426 ***	0.036-	3.97	98.98%	1.02%
Standard deviation	0.0613 **	0.0296	2.07	—	—
VTRAFWAY: Two-way, divided, median	0.2466 ***	0.0303	8.14	—	—
VTRAFWAY: One-way	0.2140 ***	0.0815	2.62	—	—
VALIGN: Straight	0.1703 ***	0.0609	2.8	—	—
REGION: Midwest	−0.1221 ***	0.0375	−3.25	—	—
REGION: South	−0.0916 ***	0.0302	−3.04	—	—
▲ Light Condition: Daylight	0.1929 ***	0.0415	4.65	—	—
$\mu_{1}$	0.5090 ***	0.0129	39.56	—	—
$\mu_{2}$	1.3173 ***	0.0243	54.17	—	—
ρ (correlation parameter)	0.2440 ***	0.0143	17.06	—	—
Final log-likelihood	−23,365				

Note: ***, **, * mean Significance at 1%, 5%, 10% level. ▲ means this variable is significant for both drivers.
